# Retraction Note: GRP78 determines glioblastoma sensitivity to UBA1 inhibition-induced UPR signaling and cell death

**DOI:** 10.1038/s41419-024-06574-0

**Published:** 2024-03-05

**Authors:** Guanzheng Liu, Jiefeng Yu, Runqiu Wu, Lin Shi, Xu Zhang, Wanhong Zhang, Xiaomin Zhong, Yifeng Wang, Huan Li, Yang Shen, Changyong Wu, Rutong Yu, Mingshan Niu, Xuejiao Liu

**Affiliations:** 1 Insititute of Nervous System Diseases, The Affiliated Hospital of Xuzhou Medical University, Xuzhou Medical University, Xuzhou, Jiangsu China; 2 Department of Neurosurgery, The Affiliated Hospital of Xuzhou Medical University, Xuzhou, Jiangsu China; 3 Department of Neurosurgery, Henan Provincial People’s Hospital, People’s Hospital of Zhengzhou University, Zhengzhou, Henan China; 4 Department of general surgery, The Second Affiliated Hospital of Xuzhou Medical University, Xuzhou, Jiangsu China; 5 Department of Neurosurgery, Kaifeng Central Hospital, Kaifeng, Henan China; 6 Department of Medical Oncology, Huai’an First People’s Hospital, Nanjing Medical University, Huai’an, Jiangsu China; 7 Blood Diseases Institute, The Affiliated Hospital of Xuzhou Medical University, Xuzhou Medical University, Xuzhou, Jiangsu China

**Keywords:** CNS cancer, Cancer stem cells

Retraction to: *Cell Death and Disease* (2021) 12:733 10.1038/s41419-021-04023-w, published online 23 July 2021

The Editors-in-Chief have retracted this article after concerns were reported about some of the data presented. After publication, it was identified that the Western blot image for the β-actin control in Fig. 6B had been re-used in Fig. 5J. Areas of apparent overlap were also identified in some the flow cytometry plots presented in the supplementary materials. The Editors-in-Chief therefore no longer have confidence in the reliability of their conclusions. The authors did not respond to correspondence from the publisher about this retraction.

